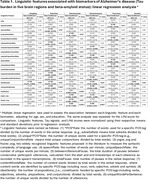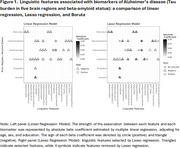# Validating Digital Linguistic Features as Potential Biomarkers of Alzheimer's Disease

**DOI:** 10.1002/alz70856_098414

**Published:** 2025-12-24

**Authors:** Haochun Huang, Zexu Li, Christina B. Young, Viktorija Smith, Ting Fang Alvin Ang, Vijaya B. Kolachalama, Rhoda Au, Jinying Chen

**Affiliations:** ^1^ Bioinformatics Program, Faculty of Computing and Data Science, Boston University, Boston, MA, USA; ^2^ Dept of Anatomy & Neurobiology, Boston University Chobanian & Avedisian School of Medicine, Boston, MA, USA; ^3^ Department of Neurology and Neurological Sciences, Stanford University School of Medicine, Stanford, CA, USA; ^4^ Framingham Heart Study, Boston University Chobanian & Avedisian School of Medicine, Boston, MA, USA; ^5^ Slone Epidemiology Center, Boston University Chobanian & Avedisian School of Medicine, Boston, MA, USA; ^6^ Department of Anatomy & Neurobiology, Boston University Chobanian & Avedisian School of Medicine, Boston, MA, USA; ^7^ Section of Computational Biomedicine, Department of Medicine, Boston University School of Medicine, Boston, MA, USA; ^8^ Computing & Data Sciences, Boston University, Boston, MA, USA; ^9^ Department of Neurology, Boston University Chobanian & Avedisian School of Medicine, Boston, MA, USA; ^10^ Department of Epidemiology, Boston University School of Public Health, Boston, MA, USA; ^11^ Biomedical Genetics, Department of Medicine, Boston University Medical School, Boston, MA, USA; ^12^ Department of Medicine/Section of Preventive Medicine and Epidemiology, Boston University Chobanian & Avedisian School of Medicine, Boston, MA, USA; ^13^ Data Science Core, Boston University Chobanian & Avedisian School of Medicine, Boston, MA, USA

## Abstract

**Background:**

Speech features derived from verbal responses to cognitive tests have been shown to indicate mild cognitive impairment and later risk of Alzheimer's Disease (AD). However, research in validating speech features using established AD biomarkers remains limited. This study aims to validate linguistic features from digital audio recording against AD biomarkers.

**Method:**

We analyzed data from participants of the Framingham Heart Study who were cognitively intact and had (1) Tau PET and amyloid PET scans conducted on the same date or within a 3‐month interval; (2) a logical memory delayed recall (LMd) test within one year of the Tau PET scan; and (3) manually transcribed response from the LMd test. Using natural language processing methods and spaCy Python library, we extracted 52 linguistic features from transcribed LMd responses, which included measures of lexical density, syntactic complexity, and speech fluency. We validated these features against AD biomarkers: (1) beta‐amyloid status (+/‐) derived through Gaussian mixture modeling and (2) Tau PET signals in five brain regions: amygdala, entorhinal, inferior parietal (IP), inferior temporal (IT), and precuneus. First, we used multiple linear regression to assess the association between each feature (dependent variable) and the biomarker status (independent variable), adjusting for age, sex, and education. Features associated with an AD biomarker (*p* <0.05) were further analyzed using Lasso regression.

**Result:**

Data from 238 participants (age: 54.9±8.3, 51.3% females, 7.1% were amyloid positive) were analyzed. While the standardized LMd test score showed no association with AD biomarkers, several linguistic features demonstrated significant associations with these biomarkers (Table 1, Figure 1). Decreased content complexity (i.e., lower ideaDensity) was associated with amyloid positivity (Beta: ‐0.542, *p* = 0.03) and higher Tau burden in entorhinal (Beta:‐0.148, *p* = 0.03), IT (Beta:‐0.143, *p* = 0.04), and Amygdala (Beta:‐0.139, *p* = 0.04). Reduced syntactic complexity (i.e., lower Yngve_avg scores) were associated with higher Tau burden in IP (Beta:‐0.168, *p* = 0.02), and IT (Beta:‐0.145, *p* = 0.04). Longer between‐utterance pause duration was positively associated with higher Tau burden in IP (Beta:0.322, *p* <0.001) and IT (Beta:0.196, *p* = 0.01).

**Conclusion:**

Linguistic‐based speech features are associated with amyloid positivity and tau accumulation and can potentially serve as digital biomarkers for preclinical AD but need further validation.